# Using Whole Genome Sequencing in an African Subphenotype of Myasthenia Gravis to Generate a Pathogenetic Hypothesis

**DOI:** 10.3389/fgene.2019.00136

**Published:** 2019-03-01

**Authors:** Melissa Nel, Nicola Mulder, Tarin A. Europa, Jeannine M. Heckmann

**Affiliations:** ^1^Neurology Research Group, Division of Neurology, Department of Medicine, University of Cape Town, Cape Town, South Africa; ^2^Computational Biology Division, Institute of Infectious Disease and Molecular Medicine, University of Cape Town, Cape Town, South Africa

**Keywords:** myasthenia gravis, African, whole genome sequencing, extraocular muscle, ophthalmoplegia, *HLA-DPB1*, extreme phenotype, association

## Abstract

Myasthenia gravis (MG) is a rare, treatable antibody-mediated disease which is characterized by muscle weakness. The pathogenic antibodies are most frequently directed at the acetylcholine receptors (AChRs) at the skeletal muscle endplate. An ophthalmoplegic subphenotype of MG (OP-MG), which is characterized by treatment resistant weakness of the extraocular muscles (EOMs), occurs in a proportion of myasthenics with juvenile symptom onset and African genetic ancestry. Since the pathogenetic mechanism(s) underlying OP-MG is unknown, the aim of this study was to use a hypothesis-generating genome-wide analysis to identify candidate OP-MG susceptibility genes and pathways. Whole genome sequencing (WGS) was performed on 25 AChR-antibody positive myasthenic individuals of African genetic ancestry sampled from the phenotypic extremes: 15 with OP-MG and 10 individuals with control MG (EOM treatment-responsive). Variants were called according to the Genome Analysis Toolkit (GATK) best practice guidelines using the hg38 reference genome. In addition to single variant association analysis, variants were mapped to genes (±200 kb) using VEGAS2 to calculate gene-based test statistics and HLA allele group assignment was inferred through “best-match” alignment of reads against the IMGT/HLA database. While there were no single variant associations that reached genome-wide significance in this exploratory sample, several genes with significant gene-based test statistics and known to be expressed in skeletal muscle had biological functions which converge on muscle atrophy signaling and myosin II function. The closely linked *HLA-DPA1* and *HLA-DPB1* genes were associated with OP-MG subjects (gene-based *p* < 0.05) and the frequency of a functional A > G SNP (rs9277534) in the *HLA-DPB1* 3′UTR, which increases *HLA-DPB1* expression, differed between the two groups (*G*-allele 0.30 in OP-MG vs. 0.60 in control MG; *p* = 0.04). Furthermore, we show that rs9277534 is an *HLA-DBP1* expression quantitative trait locus in patient-derived myocytes (*p* < 1 × 10^−3^). The application of a SNP to gene to pathway approach to this exploratory WGS dataset of African myasthenic individuals, and comparing dichotomous subphenotypes, resulted in the identification of candidate genes and pathways that may contribute to OP-MG susceptibility. Overall, the hypotheses generated by this work remain to be verified by interrogating candidate gene and pathway expression in patient-derived extraocular muscle.

## Introduction

Myasthenia gravis (MG) is a rare, but treatable antibody-mediated disease which results in fatigable weakness of skeletal muscles, including extraocular (or eye) muscles. In most individuals this is a result of pathogenic antibodies targeting the acetylcholine receptors (AChR) at the neuromuscular junction, which cause activation of complement at the muscle endplate and consequent muscle damage (Engel et al., 1977).

Though the incidence of AChR-antibody positive MG in sub-Saharan Africa is similar to global figures (Mombaur et al., 2015), and the response to MG therapies overall is similar among populations (Heckmann et al., 2007), we have recognized a subphenotype of treatment-resistant ophthalmoplegia, or OP-MG, among a subset of MG subjects of African genetic ancestry (Heckmann et al., 2007; Heckmann and Nel, 2017). This OP-MG subphenotype is characterized by severe, persistent extraocular muscle (EOM) weakness and commonly affects subjects with juvenile onset, but otherwise characteristic AChR-antibody positive MG (i.e., generalized muscle weakness which responds to treatment). The pathogenesis of the OP-MG subphenotype remains unknown though we hypothesize that individuals who develop this subphenotype may harbor African susceptibility variants which impact on the MG disease process in the particular context of the EOMs.

A previous extended whole exome sequencing (WES) study of OP-MG subjects, including untranslated region (UTR) coverage, identified a number of putative regulatory variants (Nel et al., 2017). However, this study suffered from several limitations including false positive variant calls which could not be validated by Sanger sequencing (likely PCR related) and limited coverage of the non-coding genome (which is expected to harbor a greater burden of variants contributing to complex disease risk). Here we identified a number of OP-MG associated variants in the *HLA* class II region though it was not possible to verify them with Sanger sequencing due to the complexity of this region. This was interesting because the genetic basis of MG has been investigated for more than three decades in individuals of European genetic ancestry and the consistent finding has been the association of the class I and II *HLA* region with individuals by age at MG onset (Nel and Heckmann, 2018).

The focus of the present study was to perform PCR-free whole genome sequencing (WGS) in a well characterized cohort of OP-MG and control MG individuals, all AChR antibody-positive and differing only by the responsiveness of their EOMs to standard therapy. Although the sample is small (*n* = 25), this discovery cohort represented highly selected individuals from the phenotypic extremes and matched for ancestry to maximize the power to detect association signals. Single nucleotide polymorphisms (SNPs) which were suggestive of association with OP-MG were validated in a larger cohort and a SNP to gene to pathway approach was used to prioritize genes based on skeletal muscle expression patterns.

## Materials and Methods

### Patient Samples

**FIGURE 1:**
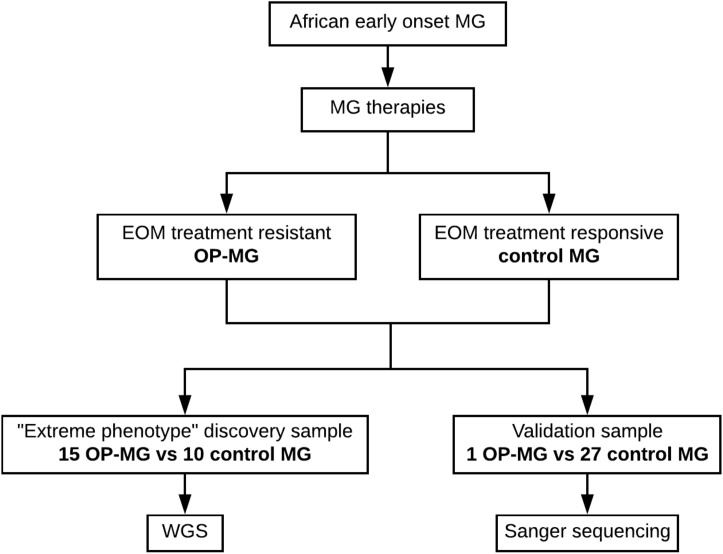
Schematic of study design showing discovery and validation samples.

Patients with generalized myasthenia gravis (MG) of early-onset (<25 years) and African genetic ancestry (either black African or Cape mixed African ancestry) were recruited for WGS. This discovery sample represented the phenotypic extremes of treatment responsivity to myasthenic-associated EOM weakness. The case group (*n* = 15) included individuals with OP-MG as previously described (Heckmann and Nel, 2017), defined as treatment resistant weakness of EOMs. The control group (*n* = 10) included individuals with no persistent EOM weakness, i.e., EOM weakness may have been present at disease presentation but responded appropriately to treatment. DNA samples from 28 African ancestry MG patients (1 OP-MG and 27 control MG) with early onset disease (<38 years) served as a validation sample to genotype selected variants. This study was approved by the UCT Faculty of Health Sciences Human Research Ethics Committee (HREC 591/2014) and all subjects gave written informed consent in accordance with the Declaration of Helsinki. The study design is outlined in Figure 1.

### DNA Extraction and Whole Genome Sequencing

Genomic DNA was extracted from buffy coats of nucleated cells obtained from anticoagulated whole blood using the salting out method (Miller et al., 1988). Sequencing libraries (2 × 150 bp read length) were prepared from DNA samples using the TruSeq PCR-free library preparation kit (Illumina). Libraries were sequenced on Illumina HiSeq sequencing instruments (30× coverage) at the Kinghorn Centre for Clinical Genomics (Sydney, Australia) and the Centre for Genomic Regulation (Barcelona, Spain).

### Read Alignment and Variant Calling

Paired end sequencing reads (FASTQ files) were aligned to the hg38 reference genome (including HLA contigs) using BWA MEM v0.7.15 (1000 Genomes Project Consortium et al., 2012) to generate BAM files. The Genome Analysis Toolkit best practice guidelines for germline SNPs and Indels were followed (GATK v3.7) (Van der Auwera et al., 2013) including duplicate read removal and base quality score recalibration of BAM files followed by variant calling using Haplotypecaller (first on individual samples to generate GVCF files and then on the entire cohort to generate a final multisample VCF file) (Poplin et al., 2017). Variant quality score recalibration (VQSR) was performed separately for SNPs and Indels using a tranche sensitivity threshold of 99% to remove false positive calls. Variants were annotated using the Ensembl variant effect predicter (McLaren et al., 2016).

### HLA Allele Determination

Reads aligning to the HLA region on chromosome 6 (33,064,568–33,080,777) and to the HLA contigs were extracted from the BAM files and realigned to reference sequences from the IMGT/HLA database (v3.29.0.1, 2017). HLA allele group assignment was inferred through “best-match” alignment of reads against the IMGT/HLA alleles using HLA Explore Software (Omixon).

### Case Control Association Analysis

Autosomal, bi-allelic variants were extracted from the VCF file and PLINK v1.9 (Chang et al., 2015) was used to perform various quality control procedures prior to association testing. Variant level filtering (excluding variants with MAF <5%, call rate <95% and Hardy–Weinberg (HW) equilibrium *p*-value < 1 × 10^−6^ in controls) and sample level filtering (excluding individuals with outlying missing genotype or heterozygosity rates) was performed according to previously described guidelines (Anderson et al., 2010; Reed et al., 2015). To exclude any large-scale differences in ancestry between the OP-MG and control MG groups (which could confound the case-control association analysis), principal component analysis of 50 357 variants was performed after LD based SNP pruning using PLINK v1.9 (–indep-pairwise 1000 50 0.15). The allelic association of each marker with OP-MG was tested using Fisher’s exact test (considering the unpruned dataset) and the genomic inflation estimate (lambda) was calculated for the unadjusted model based on median chisq.

### Gene and Pathway Based Analyses

As a complimentary approach to single variant association analysis, VEGAS2 was used to calculate gene (Mishra and Macgregor, 2015) and pathway (Mishra and MacGregor, 2017) based *p*-values. This software tool maps SNPs to genes based on their genomic location. We performed two analyses in parallel: stringent mapping (including SNPs within a gene plus any SNPs outside of the gene with *r*^2^ > 0.8 with SNPs within the gene) and less stringent mapping (including SNPs within a gene plus any SNPs 200 kb upstream and downstream of the 5′UTR and 3′UTR boundaries). For each mapping approach, the *p*-values from each mapping SNP are aggregated accounting for the linkage disequilibrium (LD) between SNPs and correcting for the gene size (number of SNPs). To compute pathway-based test statistics, the gene-based test statistics for gene lists in curated pathways (multiple sources including BIOCARTA, REACTOME and KEGG databases) and custom pathways (mined from various sources of EOM gene expression data, Supplementary Table S2) are aggregated and corrected for pathway size bias.

### Sanger Sequence Verification of Variants

Two variants were verified by Sanger sequencing in a validation sample of myasthenics with African genetic ancestry consisting of 1 OP-MG subject and 27 control MG subjects using the following primers: CCAGGCTGAGAGACAAAGCAGACC forward and CGTACTTATGTGCCACACAAGAC reverse for rs16834631 in *FAM92A1* and GATGGAGCTTCCGGAAGTCTTGG forward and CAAGGCAACTGCCTCTCTGCACC reverse for rs7816955 in *PEF1*.

### Cell Cultures

Dermal fibroblasts from 10 OP-MG and 5 control MG individuals were obtained from skin punch biopsies using the explant method. These were transduced with an RGD fiber modified adenovirus containing a human MyoD transgene as previously described (Nel et al., 2019). Briefly, transduced fibroblasts were maintained in differentiation medium (DMEM + 5% horse serum + 1% P/S) for 48 h to induce myogenic transdifferentiation and generate myocytes. Myocytes stained positively for sarcomeric myosin and successful myogenic transdifferentiation was further confirmed by demonstrating muscle-specific gene expression in myocytes (*CHRNA1*, *MYOD1*, and *MYOG*). Importantly, based on muscle-specific gene expression levels, the degree of myogenic transdifferentiation was similar in both OP-MG and control MG myocytes. To mimic MG-induced gene expression changes *in vitro*, myocytes were stimulated with 5% homologous MG sera for 24 h before harvesting RNA. Sera samples were sourced from AChR antibody-positive, treatment-naive MG patients with generalized myasthenia and severe extraocular muscle involvement.

### Quantitative Polymerase Chain Reaction (qPCR)

RNA was extracted from myocytes using the HighPure RNA extraction kit (Roche) according to the kit protocol. RNA concentration and purity was determined using the Nanodrop^®^ ND1000 spectrophotometer [Thermo Scientific and all ratios were within the recommended ranges (A260/280 = 1.8–2.0; A260/230 > 1.7)]. 400 ng total RNA was reverse transcribed to cDNA using the RT^2^ First Strand Kit (Qiagen) according to the manufacturer’s specifications. Quantitative PCR was performed on the cDNA samples using proprietary Quantitect primer assays (Qiagen) (*RPLP0*, *HLA-DPB1*) and RT^2^ SYBR Green Mastermix (Qiagen) on the 7900HT Fast Real-Time PCR System (Applied Biosystems). *RPLP0* was selected from a panel of 10 reference genes which were screened for their expression stability in myocytes (Nel et al., 2019). Individual data points were calculated as 2^−Δ^*^C^*^q^, where Δ*C*q = target gene *C*q – reference gene *C*q (Schmittgen and Livak, 2008).

### Data Visualization

Quantile–Quantile (Q–Q) and manhattan plots were created in R (version 3.5.1) using the qqman package (Turner, 2018). The heatmap of skeletal muscle tissue RNAseq expression data from the Genotype-Tissue Expression (GTEx) project was generated using the GTExPortal [1]. Graphs of qPCR expression data were created using Prism 7 (version 7.0c).

### Computation

Computations were performed using facilities provided by the University of Cape Town’s ICTS High Performance Computing team: hpc.uct.ac.za and the Bioinformatics Unit at the Centre for Genomic Regulation (CRG), Barcelona.

## Results

### Clinical Characteristics of Study Participants

The clinical characteristics of the study participants are summarized in Table 1. For the WGS discovery sample, all subjects had early onset MG and there was no significant difference in the age of disease onset between OP-MG and control MG groups (14 years vs. 16 years, *p* = 0.450), or the sex ratios (*p* = 0.13). The WGS sample comprised 11 black African ancestry individuals (44%) and 14 Cape mixed African ancestry (M/A) individuals (56%) with similar ancestry proportions in OP-MG and control MG groups. While the sex and ancestry proportions were similar between the WGS and validation samples, the age at MG onset was significantly higher in the validation sample compared to the WGS sample (*p* = 4 × 10^−8^). This was primarily because we tried to reduce confounders for the highly selected sample undergoing WGS by matching for age which was previously identified as a biological factor.

**Table 1:** Demographic characteristics of the study participants.

	WGS sample		*P*-value	Validation sample	*P*-value
Clinical	OP-MG	Control MG	OP-MG vs. control MG	1 OP-MG + 27 control MG	WGS vs. validation
characteristics	(*n* = 15)	(*n* = 10)		(*n* = 28)	sample
Age at disease onset yrs (IQR)	14 (11–17)	16 (15–22)	0.450	26 (22–31)	4 × 10^−8^
**Sex**					
Female *n* (%)	9 (60)	9 (90)	0.131	23 (82)	0.404
Male *n* (%)	6 (40)	1 (10)		5 (18)	
**Ancestry**					
Black African *n* (%)	8 (53)	3 (30)	0.288	6 (21)	0.091
Cape mixed African *n* (%)	7 (47)	7 (70)		22 (79)	

*IQR, interquartile range.*

### Description of Variants

The final VQSR filtered callset contained ∼18 million variants, including ∼2 million (11%) novel variants, with an overall Ti/Tv ratio of 2.12 and a heterozygous/homozygous ratio of 2.06 which is in line with previously published genome-wide quality control metrics, particularly for African datasets (DePristo et al., 2011; Guo et al., 2014). A high proportion of the detected variants were singletons 29% (∼5 million). Overall there were ∼5 million variants per genome which is consistent with previously published data for African populations (Auton et al., 2015).

### Population Structure

**FIGURE 2:**
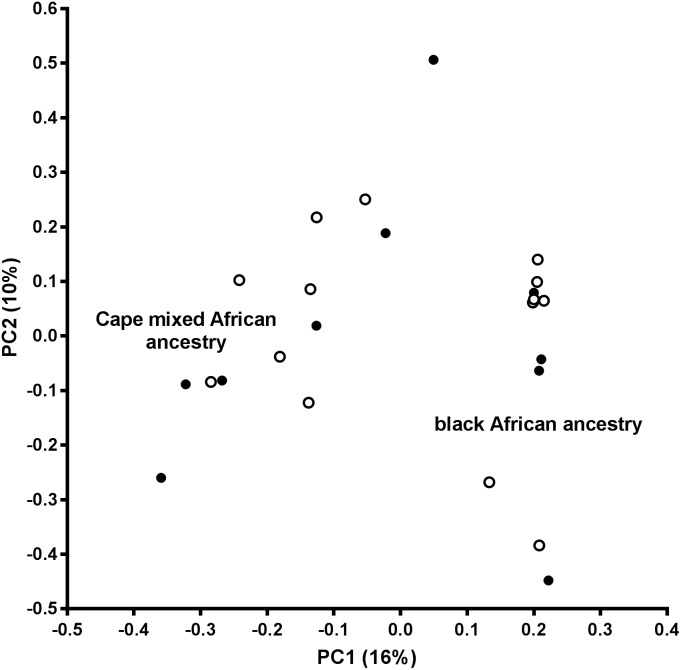
Principal component analysis (PCA) plot. PC1, principal component 1; PC2, principal component 2; open circles, OP-MG individuals; closed circles, control MG individuals.

An assessment of the population structure within the dataset was investigated using principal component analysis (PCA) after LD based SNP pruning. Combined, principal components 1 and 2 explain 26% of the total variance within the dataset; these are visualized on the PCA plot shown in Figure 2. The samples segregate into two clusters reflecting the black African and Cape M/A groups. Present day South Africans include a major ethnolinguistic group of black African South-Eastern Nguni-language (isXhosa and isiZulu) speakers. The Cape M/A ancestry population (predominantly Khoisan and Nguni-speaking African ancestry as well as smaller genetic contributions from Europeans and Southeast Asians) (De Wit et al., 2010; Quintana-Murci et al., 2010) comprise the most prevalent sub-population in the Western Cape region where this study was conducted. Despite their shared African ancestry with the black African ancestry individuals, the Cape M/A ancestry individuals form a dispersed but distinct cluster reflecting the admixed nature of this population which has considerable ancient African hunter-gatherer (Khoisan) and lesser non-African genetic contributions (Choudhury et al., 2017). Two out of the three outlier samples in the black African ancestry cluster represent individuals from other African countries (Zimbabwe and Burundi). Importantly, OP-MG and control MG individuals are equally represented in both ancestry groups which indicates that the case control association analysis will not be confounded by differences in population structure.

### Analysis of WGS Data to Identify Association Signals

**FIGURE 3:**
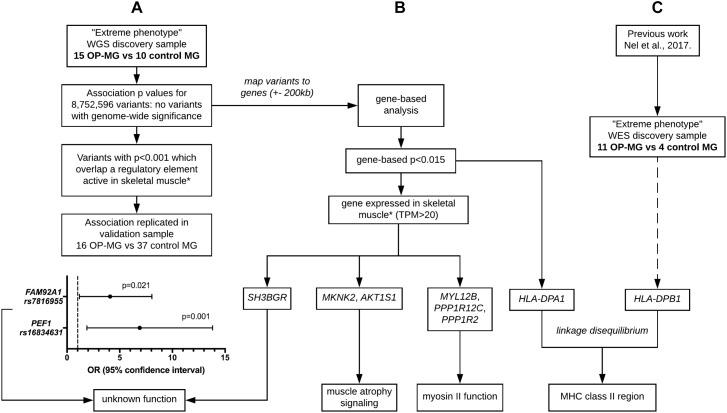
Outline of analysis approaches applied to whole genome sequencing data. **(A)** Single variant association analysis. **(B)** Gene-based association analysis. **(C)** HLA region association analysis. ^∗^ skeletal muscle expression data used for tissue-based prioritization since extraocular muscle data not available. Extreme phenotype refers to individuals with either severe EOM weakness (OP-MG cases) or no EOM weakness (MG controls). WGS, whole genome sequencing. WES, whole exome sequencing. MHC, major histocompatibility complex.

Various approaches were used in parallel (outlined in Figure 3) to identify OP-MG associated variants and genes. The results of these analyses were interpreted in conjunction with our previous work, involving WES of OP-MG and control MG subjects (Nel et al., 2017), to collectively generate hypotheses regarding OP-MG susceptibility pathways.

#### Single Variant Association Analysis

**FIGURE 4:**
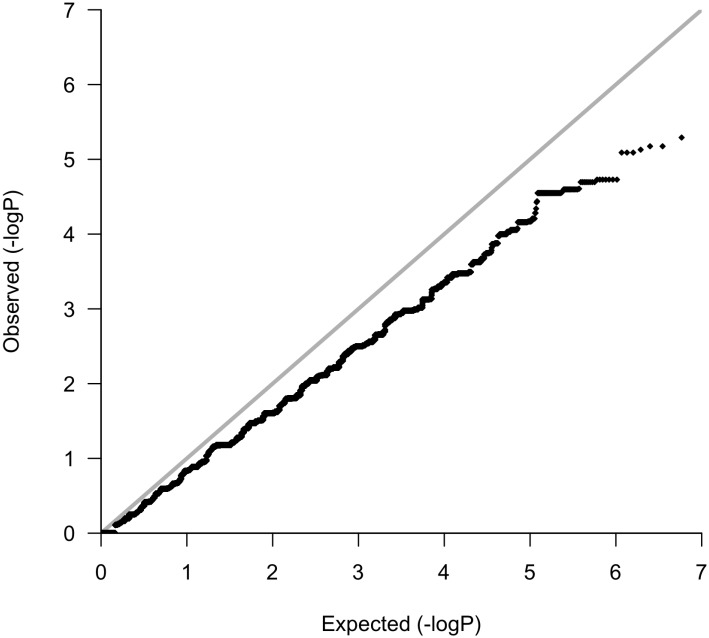
Quantile–quantile plot.

Following variant and sample level filtering, the frequency of 8,752,596 variants were compared between case and control groups (i.e., OP-MG vs. control MG) using Fisher’s exact test (Figure 3A). The black points in the quantile–quantile (Q–Q) plot in Figure 4 show the observed p values (sorted from largest to smallest) plotted against the expected *p*-values from a theoretical χ^2^-distribution (Ehret, 2010). The gray straight line in the Q–Q plot indicates the distribution of SNPs under the null hypothesis. The black points form a straight line which is “deflated” relative to the gray line suggesting that the analyses were underpowered due to the small sample size in this study. Consequently, there were no variant associations which reached genome-wide significance (*p* < 5 × 10^−8^).

**FIGURE 5:**
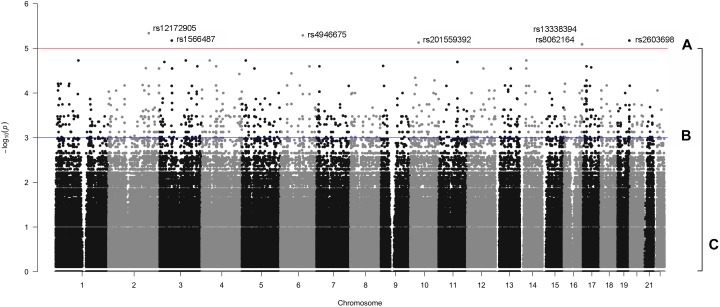
Manhattan plot displaying single variant association signals (–log_10_ *P*-value) plotted against chromosomal location. **(A)** *p* < 1 × 10^−5^, **(B)** *p* < 1 × 10^−3^, and **(C)**, all variants.

The manhattan plot shows 7 variants with suggestive association with either the OP-MG or control MG phenotype (*p* < 1 × 10^−5^, Figure 5A) which are summarized in Supplementary Table S1. Five out of 7 variants had a lower frequency in OP-MG compared to control MG and all variants are common in African populations (1000 genomes data). None of these top associated variants had any predicted functional consequences. While this may be true of many top GWAS hits in studies using chip data, where the top SNPs may not themselves be pathogenic but may “tag” other functional variants in LD, this is an unlikely scenario in our study since we have genome-wide variant coverage.

Therefore, in order to further prioritize variants with sub-genome wide significance thresholds, we screened the VEP “impact” annotations of 1,751 variants with *p* < 0.001 (Figure 5B). Seven variants were classified as “low impact” (splice region, intron and synonymous variants) and two variants were classified as “moderate impact” (missense variants) but were predicted to be benign by various prediction tools. The remainder of the variants were classified as “modifiers” and included intergenic variants and variants in up- and downstream gene regions, some of which overlapped regulatory features.

Since non-coding genetic variation was hypothesized to contribute to OP-MG susceptibility, we applied a tissue-specific prioritization approach to identify which modifier variants overlapped a regulatory feature active in muscle [human skeletal muscle myoblast and myotube (HSMM and HSMMtube) samples from the ENCODE project and psoas muscle samples from the Roadmap Epigenomics Project]. Muscle samples were chosen since there is no publically available expression data for human EOM. This analysis identified 13 variants which were more common in OP-MG compared to control MG. Two upstream gene variants, rs7816955 in *FAM92A1* (*p* = 9.2 × 10^−4^) and rs16834631 in *PEF1* (*p* = 1.5 × 10^−4^), overlapped Ensembl regulatory features classified as active promoters based on epigenome activity in relevant muscle cell lines. While the *FAM92A1* variant did not overlap any Ensembl motif features, the *PEF1* variant overlapped 24 putative transcription factor binding sites based on binding matrices, one of which was a high information position with predicted decreased binding of the RFX3::FIGLA transcription factor pair.

Both variants had a reported frequency ≤ 0.30 among African controls and ≤0.10 among European controls (1000 Genomes Project) and were validated by Sanger sequencing in the WGS sample. Their frequency was also determined in a validation sample (*n* = 28) which confirmed the association of these variants with OP-MG: *PEF1* rs16834631 0.57 in OP-MG vs. 0.16 in control MG (*p* = 0.001) and *FAM92A1* rs7816955 0.47 in OP-MG vs. 0.18 in control MG (*p* = 0.021; Figure 3A).

#### Gene-Based Association Analysis

A single variant association testing approach, while unbiased, is limited by stringent genome-wide significance thresholds which are difficult to reach after correcting for multiple testing (particularly relevant with our small sample size). Searching for association signals in single variants assumes that all affected individuals (i.e., OP-MG cases) have the same pathogenic variant(s) which does not fit with our current understanding of the genetic architecture of complex disease, which may be attributed to the joint effect of many causal loci with small effect sizes (Fu et al., 2013). To interrogate the collective biological meaning of the sub-threshold single variant associations, all variants (Figure 5C) were mapped to genes and their modest association signals were aggregated using VEGAS2 to derive gene based *p*-values (Figure 3B). A mapping threshold of 200 kb upstream and downstream of gene boundaries was chosen since this distance has been shown to increase the number of significant phenotype-pathway associations, particularly for autoimmune diseases (Brodie et al., 2016).

**FIGURE 6:**
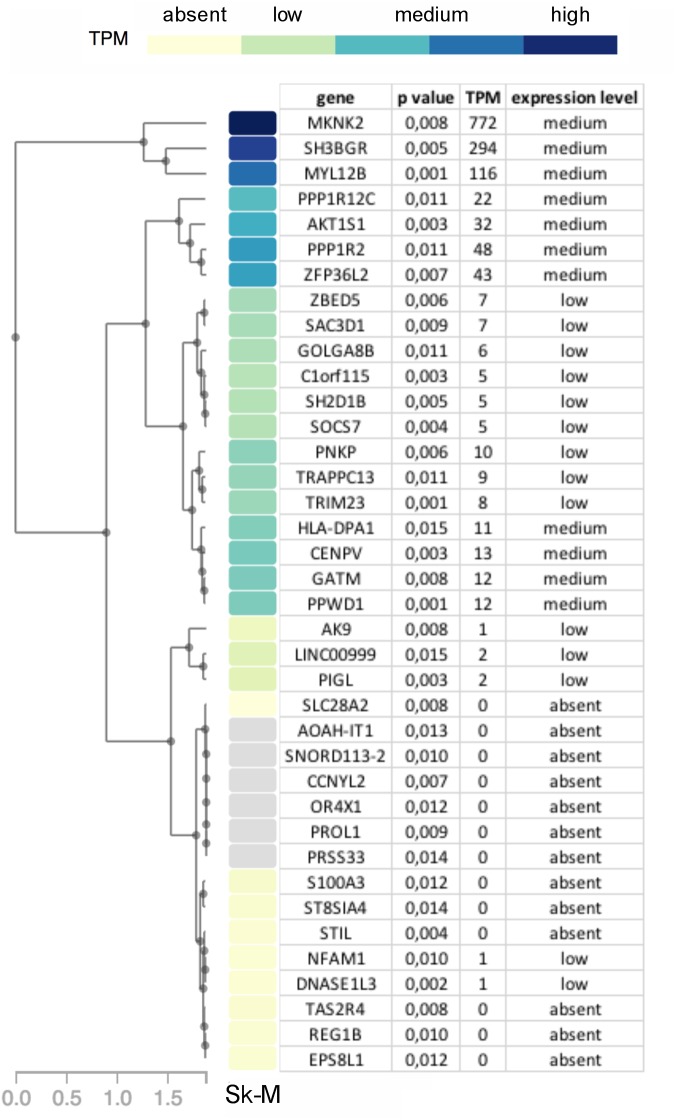
Diagram showing genes with *p*-value < 0.015 in OP-MG vs. control MG gene based association analysis with a heat map of their respective transcripts per million (TPM) expression value based on skeletal muscle tissue RNAseq expression data (Sk-M) from the Genotype-Tissue Expression (GTEx) project.

While no genes had significant *p*-values after correcting for multiple testing of 23,361 genes, 38 genes had a *p*-value ≤ 0.015. These were prioritized by determining their tissue expression using RNAseq expression data from the Genotype-Tissue Expression (GTEx) project (Aguet et al., 2017). Since there is no available expression data for the specific allotype of EOM, we prioritized genes based on their expression level in skeletal muscle tissues. Eleven genes had a medium expression level in skeletal muscle defined as a transcripts per million (TPM) value of 11–1,000 (shown in blue boxes in Figure 6). The functions of proteins encoded by genes with TPM > 20 are summarized in Table 2.

**Table 2:** Top 7 genes (based on GTEx skeletal muscle expression data, TPM > 20) with the muscle-specific function of their encoded proteins.

Gene	Function of encoded protein in skeletal muscle
*MKNK2*	Increased expression in muscle atrophy^1^ Downstream inhibitor of IGF1/Akt/mTOR hypertrophy signaling^2^
*AKT1S1*	Subunit of mTORC1 multi-protein complex
*SH3BGR*	Critical for sarcomere formation in striated muscle tissues^3^ Higher expression in mouse EOM compared to limb fibroblasts^4^
*MYL12B*	Myosin regulatory light chain which maintains the integrity of myosin I and II^5^
*PPP1R12C*	Encodes a subunit of myosin phosphatase which dephosphorylates myosin II^6^
*PPP1R2*	Binds to the catalytic subunit of protein phosphatase 1, a subunit of myosin phosphatase
*ZFP36L2*	RNA-binding protein which is downregulated during satellite cell activation^7^

*^1^Hu et al., 2012; ^2^Egerman and Glass, 2014; ^3^Jang et al., 2015; ^4^Kusner et al., 2010; ^5^Park et al., 2011; ^6^Ito et al., 2004; ^7^Galloway et al., 2016.*

#### Pathway-Based Association Analysis

The sample size was not sufficient to produce meaningful pathway-based test statistics from the VEGAS2 pathway analysis which interrogated both curated and custom pathways.

### *HLA* Region Associations

In our previous work we identified a unique “HLA signature” spanning the class II region of the MHC in OP-MG subjects (Nel et al., 2017) (Figure 3C) and the gene-based analysis in the present study also identified association signals in this region (*HLA-DPA1 p* = 0.015 and *HLA-DPB1 p* = 0.033). We therefore performed HLA typing (see section “HLA Allele Determination”) to interrogate differences in *HLA-DPA1* and *HLA-DPB1* allele frequencies between OP-MG and control groups. In our sample, *HLA-DPB1* allele diversity (12 alleles plus ambiguous alleles for 6 individuals) was higher than *HLA-DPA1* allele diversity (5 alleles) which is similar to studies in European populations (Hollenbach et al., 2012). We found differences in the frequency of 3 *HLA-DP* alleles between OP-MG and control MG (Table 3). Interestingly, for the *HLA-DPB1* locus, where alleles can be divided into two groups based on their associated HLA-DPB1 expression levels, we found that the proportion of “low expression” and “high expression” alleles differed between the OP-MG and control MG groups (*p* = 0.021). The *HLA-DPA1^∗^105:01* allele, the most common “low expression” allele in our sample and only observed in OP-MG individuals, appears to be common in African populations. The expression level of *HLA-DPB1* alleles was shown to be correlated with the genotype at rs9277534, a functional *A > G* SNP located in the 3′UTR of *HLA-DPB1* (Thomas et al., 2012). The *G*-allele of this SNP increases *HLA-DPB1* expression levels by altering the binding affinity of various microRNAs (Shieh et al., 2018). In keeping with the observed *HLA-DPB1* frequency differences (Schöne et al., 2018), we found a higher frequency of the rs9277534 *G*-allele in the control MG group.

**Table 3:** Frequency of *HLA-DPA1* and *HLA-DPB1* alleles and rs9277534 A > G in OP-MG and control MG subgroups and population controls.

*HLA-DP* alleles	OP-MG freq *n* = 15^∗^	Control MG freq *n* = 10^∗^	*p*-value OP-MG vs. control MG	African control freq^1^ *n* = 48	Caucasian control freq^1^ *n* = 75	*p*-value African vs. Caucasian control
*HLA-DPA1^∗^01:03*	15 (0.50)	3 (0.15)	*p* = 0.009	30 (0.31)	115 (0.77)	*p* < 1 × 10^−7^
*HLA-DPA1^∗^02:01*	3 (0.10)	9 (0.45)	*p* = 0.007	23 (0.24)	26 (0.17)	0.242
*HLA-DPB1^∗^105:01* (low expression^2^)	7 (0.39)	0	*p* = 0.009	25 (0.26)	2 (0.01)	*p* < 1 × 10^−7^
*HLA-DPB1* rs9277534 A > G (high expression^3^)	9 (0.30)	12 (0.60)	*p* = 0.04	789 (0.60)	317 (0.32)	*p* < 1 × 10^−7^

*^1^Goldfein, 2017; ^2^Schöne et al., 2018; ^3^Thomas et al., 2012. ^∗^For HLA-DPB1, allele assignment was ambiguous for 6 OP-MG and 3 control MG individuals and these samples were excluded from the analysis.*

**FIGURE 7:**
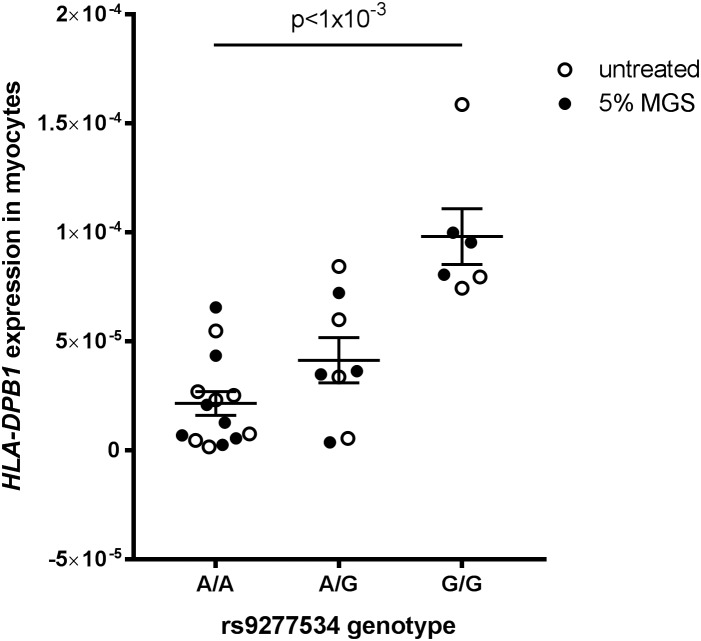
*HLA-DPB1* rs9277534 is an eQTL in myocytes. Myocytes (derived from transdifferentiated dermal fibroblasts) from OP-MG (*n* = 10) and control MG (*n* = 5) individuals were either left untreated or exposed to 5% MG sera for 24 h before RNA was harvested for analysis of gene expression by quantitative PCR. *HLA-DPB1* expression levels were determined using relative quantification (2^−Δ^*^C^*^q^) where Δ*C*_q_ represents *HLA-DPB1* Cq – RPLP0 Cq (reference gene). An ordinary one-way ANOVA was used to compare genotype groups using pooled data from 2 independent experiments (untreated myocytes and myocytes exposed to MG). Error bars show SEM. One OP-MG sample with A/A genotype was excluded as an outlier.

*HLA-DPB1* rs9277534 genotype-expression correlations have been demonstrated in blood (Yamazaki et al., 2018) but there is no data on this expression quantitative trait locus (eQTL) in skeletal muscle tissue. We therefore analyzed *HLA-DP* expression grouped by rs9277534 genotype in myocytes derived from transdifferentiated dermal fibroblasts from OP-MG and control MG subjects (Nel et al., 2019) and found that the *G*-allele increased *HLA-DPB1* expression levels (Figure 7, *p* < 1 × 10^−3^).

## Discussion

In this study we have used various strategies to mine WGS data in an attempt to generate hypotheses regarding the pathogenetic basis of a subphenotype of a rare autoimmune disease, myasthenia gravis. The subphenotype is characterized by treatment resistance of the eye muscles, or EOMs, whereas the non-ocular muscles respond to standard MG therapies (Heckmann and Nel, 2017). EOM is a specific allotype of muscle tissue because it differs from limb muscles in many respects (Porter et al., 2001). Since only a proportion of MG subjects develop the OP-MG subphenotype, the pool of affected individuals available for genetic studies is small. Nonetheless, we employed a focused strategy using extreme subphenotype sampling of OP-MG cases vs. MG disease controls to perform a genome wide analysis. Putative OP-MG susceptibility variants, genes and pathways were identified following prioritization based on known tissue-specific expression patterns in skeletal muscle since gene expression data for EOM is not available.

We have identified three main candidate pathogenic themes which we postulate are involved in developing OP-MG, and preliminary functional studies show at least some support for these hypotheses. Briefly, we summarize evidence gleaned from other areas, using the principle of triangulation, to lend support to the generated hypotheses.

The first two themes relate to muscle atrophy and muscle recovery/remodeling. The EOMs may be more susceptible to complement-mediated muscle endplate injury during MG (in Soltys et al., 2008) due to their relatively lower expression levels of complement regulatory proteins, particularly decay accelerating factor (DAF) (Kaminski et al., 2004). We previously screened the *DAF* gene in OP-MG subjects and found a higher frequency of a functional *DAF* promoter polymorphism compared to controls which impaired transcriptional upregulation of DAF expression in patient-derived cell lines following a lipopolysaccharide immune stimulus (Heckmann et al., 2010). Also, clinically and at surgery, the EOMs in the most severe cases of OP-MG are thin/atrophic, not fibrotic and unable to generate muscle force (Heckmann and Nel, 2017). Although there is limited histological data on EOMs in MG, neurogenic atrophy is a common pathological observation in the muscle biopsies of MG cases, (Oosterhuis and Bethlem, 1973) and likely to be the result of “functional denervation,” or the disconnection between the nerve and muscle endplate secondary to MG-induced damage (Nakano and Engel, 1993). With that in mind, the gene-based analysis identified two genes (*MKNK2*, *AKT1S1*) involved in the IGF1/AKT/mTOR pathway, which is a key pathway in promoting muscle atrophy following denervation (Tang et al., 2014) (Table 2). In keeping with these unbiased findings, our previous gene expression profiling of OP-MG myocytes using a panel of genes relevant in several MG studies found expression of genes from this pathway (*IGF1*, *AKT1*, and *AKT2*) were strongly correlated in OP-MG myocytes but not in the myocytes from control MG cases (Nel et al., 2019). Interestingly, *IGF1* is highly expressed in EOMs where it regulates both the muscle mass and force generation of these muscles, and its signaling is dysregulated in paralyzed EOMs (Altick et al., 2012).

Subsequent to MG damage we would expect the EOMs to undergo “regeneration” or remodeling due to their high numbers of resident satellite cells (McLoon and Wirtschafter, 2003), and this process requires the synthesis of new structural muscle proteins. We were therefore interested to observe that 3 of the 7 genes (*MYL12B*, *PPP1R12C*, and *PPP1R2)* identified by the gene-based analysis (Table 2) are involved in the stability and regulation of myosin II which is a prominent isoform in EOMs expressed by fast type IIA and IIB muscle fibers, respectively (Park et al., 2011).

While unbiased, genome-wide association studies (GWAS) typically employ very large samples, the application of this approach to the study of susceptibility to MG, has not been very informative in terms of identifying new disease loci. In two recent GWAS in MG, the strongest association signals identified were localized to the *HLA* region (Gregersen et al., 2012; Renton et al., 2015), which was already identified over 3 decades ago in a small case-control sample (Compston et al., 1980). The third theme we identified relates to the *HLA* region since we found an association signal with lower *HLA-DPB1* expression in OP-MG which results from a functional polymorphism in the 3′UTR of *HLA-DPB1*. Although the *HLA-DP* locus is not in LD with other *HLA* loci, the expression levels of *HLA-DPB1* are increasingly recognized to have clinical relevance (Fleischhauer, 2015). While the main MG susceptibility locus lies in the class I or II region depending on the age at symptom onset (Nel and Heckmann, 2018), *HLA-DPB1* alleles may influence the phenotypic manifestations of the MG disease process in different individuals.

We also identified association signals in *PEF1* and *FAM92A1* which were validated in an independent sample, although the functional relevance of these genes in EOM is unknown. This highlights the importance of validating the hypotheses generated by this work in patient-derived EOM tissue, preferably from OP-MG individuals. It is worth noting that candidate gene associations, such as those previously identified in the regulatory region of *DAF*, were not identified following the filtering criteria used in this study. This is likely due to the sample size constraints imposed by WGS which limits the ability to detect significant associations for low frequency variants such as *DAF −198 C > G*. This SNP had a frequency of 0.13 among the OP-MG subjects in this study (*p* = 0.119) which is comparable to the statistically significant association previously reported using a larger sample size (0.12, *p* = 0.001) (Heckmann et al., 2010), albeit with an overlap of two OP-MG samples between the two studies.

In conclusion, despite the limitations of using a small sample to mine whole genome data to generate pathogenic hypotheses in a structured yet unbiased approach, several lines of evidence suggest we have achieved our aims. The next step will be to analyze the functionality of these genes and pathways in patient-derived extraocular muscle tissue.

## Data Availability

The whole genome sequencing data on which the findings of this manuscript are based, have been deposited in the European Genome-Phenome Archive (EGA): https://www.ebi.ac.uk/ega/home and can be found under the following accession ID: EGAS00001003462. Access to the dataset is governed by a data access committee.

## Author Contributions

MN designed the myocyte model, performed the functional studies in myocytes, performed the genomic data analysis, and wrote the manuscript. NM provided computing resources, bioinformatics support, and editorial input. TE performed the qPCR experiments on ocular fibroblasts. JH conceived and designed the study, collected DNA samples and clinical data, and provided editorial input and funding support.

## Conflict of Interest Statement

The authors declare that the research was conducted in the absence of any commercial or financial relationships that could be construed as a potential conflict of interest.
