# Supplementary material for: Using Whole Genome Sequencing in an African Subphenotype of Myasthenia Gravis to Generate a Pathogenetic Hypothesis
Source: Front Genet. 2019 Mar 1;10:136. doi: 10.3389/fgene.2019.00136 (PMC6406016; doi:10.3389/fgene.2019.00136)
Supplement: Supplementary file 1 [file Table_1.DOCX]

**Supplementary table 1. Variants with suggestive association (p<1x10^-5^) with OP-MG or control MG**

| **rsID** | **location** | **OP-MG MAF** | **control MG MAF** | **African control freq^1,2^** | **Gene (location)** |
| --- | --- | --- | --- | --- | --- |
| rs12172905 G>A | chr2:190403536 | 0.04 | 0.65 | 0.20 | MFSD6 (upstream) |
| rs1566487 A>G | [chr3:56813803](https://useast.ensembl.org/Homo_sapiens/Location/View?contigviewbottom=variation_feature_variation%3Dnormal%2Cseq%3Dnormal;db=core;r=3:56813753-56813853;source=dbSNP;v=rs1566487;vdb=variation;vf=615611755) | 0.07 | 0.70 | 0.28 | ARHGEF3 (intron) |
| rs4946675 C>G | chr6:105620951 | 0.18 | 0.85 | 0.42 | lincRNA Z97206.2 (intron) |
| rs201559392 TCCAT/- | chr10:38784347-38784351 | 0 | 0.55 | 0.50 | intergenic |
| rs8062164 (C>G) | chr16:85269617 | 0.75 | 0.10 | 0.67 | GSE1 (intron) |
| rs13338394 (C>T) | chr16:85269814 | 0.75 | 0.10 | 0.62 | GSE1 (intron) |
| rs2603698 (C>T) | chr19:55098006 | 0.07 | 0.70 | 0.30 | PPP1R12C (intron) |

**Supplementary table 2. Custom pathways interrogated by VEGAS2 pathway analysis**

| Custom pathway | Observed p | Empirical p | Genes in pathway |
| --- | --- | --- | --- |
| Gene expression changes in differentiating myoblasts from mouse EOM^1^ | 0.999 | 0.989 | NID1, DLX1, SHOX2, EREG, GSTA2, TWIST1, SLC38A4, PRKCB, MYH8, MRC2, SERPINB2, IL12RB1, BLVRB, KCNN4 |
| Gene expression changes in mouse ocular fibroblasts^2^ | 0.999 | 0.597 | DDAH1, NES, FMO2, LMOD1, MYBPH, CNTN2, ACTA1, RASGRP3, VIT, CYP26B1, ACTG2, HNMT, NEB, CHRNA1, FRZB, MYL1, IGFBP2, DNER, KIF1A, RARB, MAPKAPK3, COL8A1, MASP1, TLR1, RASSF6, PLAC8, SNCA, PITX2, PDE5A, IL15, TLL1, SORBS2, NKD2, NPR3, LRRTM2, SERPINB9, CDKN1A, GSTA3, CD109, SLC35F1, LAMA2, CTGF, VNN1, PERP, SOSTDC1, IL6, AQP1, BMPER, HSPB1, SERPINE1, EPHX2, ADRB3, SNTG1, STMN2, FABP4, TNFRSF11B, PTPLAD2, ALDH1A1, SYK, PALM2, ANXA8, ACTA2, ANKRD1, ENTPD1, SLC22A18, PDE2A, PRSS23, GRIA4, MFRP, FLI1, ACVRL1, WIF1, PTPRB, GLIPR1, NTN4, CMKLR1, SMOC1, ACTC1, ALDH1A2, TLN2, MYLPF, HP, MYH3, PMAIP1, SLC1A6, CLEC11A, TNNT1, PEG3, LBP, WISP2, TNNC2, EYA2, SH3BGR |
| Gene expression changes in rat EOM following passive EAMG^3^ | 0.999 | 0.958 | KIF1B, C1QB, PTAFR, LAPTM5, DBT, CD53, RAB13, CRABP2, SOAT1, MYBPH, GRB14, FN1, CCL20, COLQ, NR1D2, SCN5A, CCR1, ST3GAL6, MASP1, KDR, CXCL9, EGF, GHR, EMB, CD14, CD74, GM2A, C2, PSMB8, SLC35B2, TINAG, ENPP3, AKAP12, ARPC1B, NOV, SQLE, JAK2, NINJ1, TNC, NEK6, COMMD3, CREM, PPIF, ADM, SPON1, MYOD1, CD44, CPT1A, IL18, APOBEC1, A2M, MGP, IGF1, SCARB1, ALOX5AP, MYH6, GPX2, EHD4, ALDH1A2, CIB1, IGSF6, CYBA, DUSP3, LGALS3BP, SECTM1, RAB3A, FXYD5, TYROBP, EMP3, PANK2, HCK, CSTB, BID, ACO2 |
| Gene expression changes in rat EOM following active EAMG^4^ | 0.995 | 0.290 | S100A9, TPM3, FMO3, CTSE, LAMB3, SMYD2, TMEM37, CHRNA1, NPPC, HDAC4, ST3GAL6, AADAC, EHHADH, BDH1, SPP1, HMP19, TINAG, FABP5, PTGDS, CXCL12, ACSL5, FOLR1, DGAT2, THRSP, ZBTB16, MGP, MGST1, DYRK2, TNFRSF19, DCT, CLDN10, ACSBG1, CES3, RASD1, CCL2, SPHK1, FASN, C3, KLK7, CEBPB, RUNX1 |

EAMG=experimentally acquired myasthenia gravis. 1. (Porter et al., 2006). 2. (Kusner et al., 2010). 3. (Zhou et al., 2014). 4. (Kaminski et al., 2016).

**References**

Kaminski, H. J., Himuro, K., Alshaikh, J., Gong, B., Cheng, G., and Kusner, L. L. (2016). Differential RNA Expression Profile of Skeletal Muscle Induced by Experimental Autoimmune Myasthenia Gravis in Rats. *Front. Physiol.* 7, 524. doi:10.3389/fphys.2016.00524.

Kusner, L. L., Young, A., Tjoe, S., Leahy, P., and Kaminski, H. J. (2010). Perimysial fibroblasts of extraocular muscle, as unique as the muscle fibers. *Invest. Ophthalmol. Vis. Sci.* 51, 192–200. doi:10.1167/iovs.08-2857.

Porter, J. D., Israel, S., Gong, B., Merriam, A. P., Feuerman, J., Khanna, S., et al. (2006). Distinctive morphological and gene/protein expression signatures during myogenesis in novel cell lines from extraocular and hindlimb muscle. *Physiol. Genomics* 24, 264–275. doi:10.1152/physiolgenomics.00234.2004.

Zhou, Y., Kaminski, H. J., Gong, B., Cheng, G., Feuerman, J. M., and Kusner, L. (2014). RNA expression analysis of passive transfer myasthenia supports extraocular muscle as a unique immunological environment. *Investig. Ophthalmol. Vis. Sci.* 55, 4348–4359. doi:10.1167/iovs.14-14422.
